# Comparing three summary indices to assess diet quality of Canadian children: a call for consensus

**DOI:** 10.3389/fnut.2024.1519829

**Published:** 2025-01-07

**Authors:** Seyedehatefeh Panahimoghadam, Paul J. Veugelers, Julia Dabravolskaj, Trudy Tran, Katerina Maximova

**Affiliations:** ^1^School of Public Health, University of Alberta, Edmonton, AB, Canada; ^2^MAP Centre for Urban Health Solutions, Li Ka Shing Knowledge Institute, St. Michael’s Hospital, Toronto, ON, Canada; ^3^Dalla Lana School of Public Health, University of Toronto, Toronto, ON, Canada

**Keywords:** dietary assessment, diet quality index, children, healthy eating, epidemiology, public health

## Abstract

**Purpose:**

The Healthy Eating Index-Canada 2015 (HEI-C 2015), Diet Quality Index-International (DQI-I), and Healthy Eating Food Index 2019 (HEFI-2019) are commonly used to summarize the quality of Canadian diets. This paper sought to compare these three diet quality indices with respect to their ability to capture diets of different quality in Canadian children and to discriminate between population subgroups.

**Methods:**

Data were collected in school-based surveys from grade 4–6 students (9–12 years old) in western Canada through 24-h dietary recall in 2016 (*n* = 336), 2018 (*n* = 454), and 2020/2021 (*n* = 909). Diet quality was assessed using HEI-C 2015, DQI-I and HEFI-2019. Agreement between the three indices was assessed using weighted Cohen’s kappa. Univariate and multivariable linear regression models assessed diet quality according to student’s sex, grade level, school material/social deprivation, and geographic region.

**Results:**

HEFI-2019 scores had the widest range, while DQI-I had the smallest. Agreement was 0.55 between HEI-C 2015 and DQI-I, 0.38 between HEI-C 2015 and HEFI-2019, and 0.29 between DQI-I and HEFI-2019. Boys and students from materially deprived areas reported diets of lower quality, irrespective of the index. There were no differences in diet quality across grade levels and geographic region. Energy consumption was associated positively with DQI-I and negatively HEFI-2019 scores.

**Conclusion:**

The three indices demonstrated fair to moderate agreement and varying ability to discriminate diet quality between different population subgroups of Canadian children. This study shows that the choice of a diet quality index affects the interpretation of results and practical considerations, yielding different conclusions with respect to the determinants of children’s diet quality. Seeking consensus on which diet quality index to use for research, policy and/or practice would help support dietary research and policy development, and promote dietary guidelines implementation.

## 1 Introduction

The quality of the diets of Canadian children is often poor as they fail to meet the recommended intakes for fruit, vegetables, and sodium, with a significant portion of their energy intake coming from high-fat, sugary foods (1–3). It is well established that consuming diets of good quality is essential for growth and physical development, whereas emerging evidence points to their importance for mental health and well-being, as well as academic performance (4–6). Good quality diets have been associated with better health outcomes, including body weights, blood pressure, metabolic syndrome, mental health and academic performance (7–9). An accurate measure of diet quality is essential to study the importance of good diet quality and to assess the effectiveness of dietary interventions.

Several indices have been used to assess the quality of the whole diet of Canadian children (8, 10, 11). The most commonly used indices are the Diet Quality Index-International (DQI-I) (10, 12, 13), Healthy Eating Index-Canada 2015 (HEI-C 2015) (11, 14, 15), and the more recently developed Healthy Eating Index-2019 (HEFI-2019) (16). While all three indices aim to summarize the quality of the whole diet using established dietary recommendations, they consider different sets of criteria and scoring systems (17), hindering the interpretation of dietary guidelines to improve diet quality. It is not clear which index best captures diet quality of different population subgroups and should be used for research, policy and/or practice to help support dietary research and policy development, and promote dietary guidelines implementation. An optimal diet quality index should have a wide range of scores to distinguish between individuals who consume diets of different quality. Additionally, a diet quality index should be independent of diet quantity and be able to effectively differentiate between different population subgroupings (e.g., gender, age, socioeconomic status (SES), rural/urban residence) (18). Indeed, while some studies found little to no difference in diet quality between girls and boys (10, 19), government reports suggest that Canadian girls consume, on average, more vegetables and fruit and less sodium, compared with boys (2, 3). Moreover, younger children consume diets of higher quality than older children (19, 20), and children from families with higher SES have healthier dietary patterns, characterized by lower consumption of energy-dense foods and higher consumption of fruit, vegetables, and dairy products, compared with their peers from lower SES households (21–23). To our knowledge, no previous studies compared these three commonly used diet quality indices with respect to their ability to capture diets of different quality. This exploratory study sought to assess the agreement between HEI-C 2015, DQI-I, and HEFI-2019, and describe the ability of each index to discriminate the diet quality of Canadian children across population subgroups.

## 2 Methods

### 2.1 Procedures

Using a repeated cross-sectional design, grade 4–6 students from 25 APPLE Schools were surveyed in 2016, 2018, and 2020/2021. APPLE Schools is an innovative school-based health promotion program introduced in 2008 and currently delivered in 93 elementary schools located in socioeconomically disadvantaged communities across western Canada. The APPLE Schools program delivers health promotion activities targeting healthy eating, physical activity, and mental health and well-being, and benefits over 30,000 children annually (24, 25). Data collection took place in schools during regular class time. Students were provided with unique usernames and passwords to access the online survey portal on their Chromebooks. In 2016 and 2018, trained research assistants travelled to schools to oversee data collection in each classroom. In 2020, data collection procedures shifted to an online mode as per COVID-19 protocols, with trained research assistants connecting to each classroom through Zoom to prompt survey questions projected on the whiteboard. A total of 441 (66%), 473 (67%), and 973 (78%) students from 6, 7, and 12 schools completed the survey in 2016, 2018, and 2020/2021, respectively. Students provided assent, while their parents or guardians provided active-information passive-permission consent. The Health Research Ethics Board of the University of Alberta (Pro00061528) and the school boards that participated in the study approved all study procedures.

### 2.2 Measures

Students completed an interactive web-based 24-h dietary recall tool to derive diet quality (26). The tool has been previously validated in youth and prompts children to report all food and beverage items consumed the previous day, providing portion size images for each item and other cues to help students recall their intake. The tool was administered in springtime on Tuesdays, Wednesdays, Thursdays or Fridays, so that all collected dietary data pertained to the intake on springtime weekdays. Student responses were analyzed using nutrient databases (Canadian Nutrient File [CFN], Elizabeth Stewart Hands and Associates [ESHA], U.S. Department of Agriculture [USDA]) to yield daily intake data for 6 food groups (i.e., vegetables, fruits, grain products, meat and alternatives, milk and alternatives, and other), energy intake (i.e., total caloric intake, caloric intake from fat and saturated fats), 10 macronutrients and 23 micronutrients (26). To control for false reporting, students reporting implausible values of energy intake <500 or > 5000 kcal (*n* = 91 [2016], 17 [2018], 64 [2020/21]) were excluded from analysis (27).

Diet quality was assessed using three dietary indices.

#### 2.2.1 Healthy Eating Index-Canada 2015

HEI was initially developed by the United States Department of Agriculture in 1995 (28–30). It is designed to reflect the recommendations outlined in the Dietary Guidelines for Americans and to promote healthy eating patterns by assessing two key categories of the diet: adequacy and moderation. HEI was adapted for use in Canada in 1995, 2005, 2010, and 2015 (31–35). In this study, HEI-C 2015 was calculated using the Canada’s Food Guide 2007 recommendations for 9-13-year-old children (36). HEI scores range from 0 to 100, with higher scores indicating better diet quality.

#### 2.2.2 Diet Quality Index-International

DQI-I is an international index developed in 2003 (37). It provides flexibility in regards to the components of a healthy diet included in the index calculation and hence enables comparison of dietary patterns across countries. By incorporating both foods and nutrients in diet quality evaluation, DQI-I takes into account the diversity of food consumption across different countries. DQI-I measures the four key categories of dietary intake: variety, adequacy, moderation, and overall balance. DQI-I scores range from 0 to 100, with higher scores indicating better diet quality.

#### 2.2.3 Healthy Eating Food Index-2019

HEFI-2019 was developed by Health Canada in 2022 to assess adherence to the new Canada’s Food Guide 2019 dietary recommendations among Canadians aged two years and older (16). HEFI-2019 assesses the intake of 10 specific dietary components, including five foods and four nutrients, with one component measuring the beverage intake. HEFI-2019 scores range from 0 to 80, with higher scores indicating better diet quality. To facilitate comparisons with DQI-I and HEI-C 2015, HEFI-2019 scores were multiplied by 1.25 to range from 0 to 100.

Supplementary Material details how each index is constructed. Full details are provided here (16, 28, 37).

#### 2.2.4 Student- and school-level characteristics

Students reported their sex (girl vs. boy) and grade (4–6). Geographic region (rural vs. urban) and school material and social deprivation were derived from 2016 Canada Census data based on schools’ postal codes (38, 39). Higher quintiles of material and social deprivation indices indicate higher deprivation. To ensure sufficient number of schools in each group of materially and socially deprived areas, quintiles 1–3 vs. 4–5 of the material deprivation index and quintiles 1–2 vs. 3–5 of the social deprivation index were combined.

### 2.3 Data analyses

The properties of each index were described using means, standard deviations (SD), minimum to maximum ranges, and coefficients of variation (CV). Simple linear regression was used to assess differences in diet quality indices measured at three time points. Percent agreement and weighted Cohen’s kappa coefficients were used to assess the level of agreement between the three indices. Total scores were categorized into quartiles since no cut-off points for differentiating good vs. poor diet quality have been previously proposed for HEI-C 2015 and HEFI-2019. The weighted Cohen’s kappa coefficients were calculated since more than two categories for each index were being compared (40). Quadratic weighting was used to account for the severity of disagreements (whereas unweighted kappa treats all disagreements equally). Next, it was assessed whether sex, grade level, energy intake, material and social deprivation quintiles, and geographic region are associated with each of the three indices, by adding these variables singularly and simultaneously to the univariate and multivariable linear regression models, respectively. The F tests, adjusted R-Squared, and root mean square deviation (RMSD) were used to assess the goodness of fit of the multivariable linear regression models. Students with missing values on sex and/or grade level were excluded (*n* = 14 [2016], 2 [2018], 0 [2020/21]). All statistical analyses were conducted using Stata 17.0 (College Station, TX). A *p*-value <0.05 was considered statistically significant.

## 3 Results

Data from 336 (2016), 454 (2018), and 909 (2020/21) students were available for analysis. Student and school characteristics are shown in Table 1. Over half (51.4%) were girls. About one-third were in grade 4, one-third in grade 5, and one-third in grade 6 (30.6, 37.1, 32.3%, respectively). Of 25 participating schools, 60.8% were located in urban areas. HEI-C 2015 and DQI-I had similar average scores and trends over time in each cross-sectional sample. Between 2016 and 2020/2021 HEI-C 2015 declined from 54.7 (SD = 13.9) to 49.5 (SD = 12.9), and DQI-I from 55.6 (SD = 9.7) to 53.2 (SD = 9.9). The HEFI-2019 scores were markedly lower than HEI-C 2015 and DQI-I in each cross-sectional sample and showed little variation over time: 45.0 (SD = 13.9) in 2016, 44.7 (SD = 13.1) in 2018, and 44.9 (SD = 13.6) in 2020/2021 (Table 1). Overall, the distribution of DQI-I scores had the lowest variability, ranging from 19.9 to 83.6 (CV = 18.1%), compared to HEI-C 2015 that ranged from 11.7 to 95.3 (CV = 25.8%) and HEFI-2019 from 8.6 to 90.1 (CV = 30.1%), with the latter having the widest range of scores.

**Table 1:** School and participant characteristics in 2016, 2018, and 2021.

Student characteristics	2016 (*n* = 336)	2018 (*n* = 454)	2021 (*n* = 909)	Total (*n* = 1699)
Sex, *n* (%)				
Girls	181 (53.9)	225 (49.6)	468 (51.5)	874 (51.4)
Boys	155 (46.1)	229 (50.4)	441 (48.5)	825 (48.6)
Grade, *n* (%)				
4	93 (27.7)	141 (31.1)	286 (31.5)	520 (30.6)
5	119 (35.4)	175 (38.6)	336 (37.0)	630 (37.1)
6	124 (36.9)	138 (30.4)	287 (31.6)	549 (32.3)
Diet quality index, mean (SD)				
HEI-C 2015	54.7 (13.9)	52.4 (12.9)	49.5 (12.9)	51.3 (13.2)^b^
DQI-I	55.6 (9.7)	55.0 (9.4)	53.2 (9.9)	54.1 (9.8)^b^
HEFI-2019^a^	45.0 (14.0)	44.7 (13.1)	45.0 (13.6)	44.9 (13.5)
Diet quality index, range (CV%)				
HEI-C 2015	19.3–93.4 (25.4)	14.6–95.3 (24.6)	11.7–89.3 (26.1)	11.7–95.3 (25.7)
DQI-I	28.0–80.8 (17.4)	26.1–80.6 (17.1)	19.9–83.9 (18.6)	19.9–83.9 (18.1)
HEFI-2019^a^	8.6-84.0 (31.1)	13.5–82.7 (29.3)	10.5–90.1 (30.2)	8.6–90.1 (30.1)

School characteristics	2016 (*n* = 6)	2018 (*n* = 7)	2021 (*n* = 12)	Total (*n* = 25)
Material deprivation quintile, *n* (%)				
1 (least deprived)	0 (0)	1 (14.3)	2 (16.7)	3 (12.0)
2	1 (16.7)	1 (14.3)	3 (25.0)	5 (20.0)
3	0 (0)	0 (0)	4 (33.3)	4 (16.0)
4	0 (0)	3 (42.9)	3 (25.0)	6 (24.0)
5 (most deprived)	5 (83.3)	2 (28.6)	0 (0)	7 (28.0)
Social deprivation quintile, *n* (%)				
1 (least deprived)	1 (16.7)	2 (28.6)	3 (25.0)	6 (24.0)
2	2 (33.3)	0 (0)	4 (33.3)	6 (24.0)
3	1 (16.7)	2 (28.6)	2 (16.7)	5 (20.0)
4	1 (16.7)	3 (42.9)	1 (8.3)	5 (20.0)
5 (most deprived)	1 (16.7)	0 (0)	2 (16.7)	3 (12.0)
Geographic region, *n* (%)				
Urban	0 (0)	0 (0)	3 (25.0)	3 (12.0)
Rural	6 (100)	7 (100)	9 (75.0)	22 (88.0)

CV, coefficient of variation; SD, standard deviation; HEI-C 2015, Healthy Eating Index-Canada 2015; DQI-I, Diet Quality Index-International; HEFI-2019, Healthy Eating Food Index 2019.

a HEFI-2019 scores have been recalibrated from a maximum of 80 to a maximum of 100 by multiplying the scores by 1.25.

b *p-value for trend from simple linear regression is < 0.0001.*

Percent agreement and weighted kappa scores varied across the survey years but were statistically significant for all comparisons (Table 2). In a combined sample of students who participated in any of the survey cycles, percent agreement between HEI-C 2015 and DQI-I was 0.88 (95% Confidence Interval [CI]: 0.87, 0.89), between HEI-C 2015 and HEFI-2019 – 0.83 (95% CI: 0.82, 0.84), and between DQI-I and HEFI-2019 – 0.80 (95% CI: 0.79, 0.81). For this combined sample, weighted Cohen’s kappa coefficient for agreement between HEI-C 2015 and DQI-I was 0.55 (95% CI: 0.52, 0.59), between HEI-C 2015 and HEFI-2019 0.38 (95% CI: 0.35, 0.42), and between DQI-I and HEFI-2019 0.29 (95% CI: 0.25, 0.33). These values of weighted Cohen’s kappa coefficients translate into fair to moderate agreement (41).

**Table 2:** Percent agreement and weighted Cohen’s kappa coefficients (95% CI) for HEI-C 2015, DQI-I, and HEFI-2019.

		HEI-C 2015		DQI-I	
		Percent agreement (95% CI)	Cohen’s kappa coefficient (95% CI)	Percent agreement (95% CI)	Cohen’s kappa coefficient (95% CI)
2016	DQI-I	0.89 (0.88, 0.91)	0.62 (0.55, 0.69)	n/a	n/a
	HEFI-2019^a^	0.83 (0.81, 0.86)	0.42 (0.34, 0.49)	0.81 (0.78, 0.84)	0.33 (0.25, 0.41)
2018	DQI-I	0.88 (0.86, 0.89)	0.55 (0.49, 0.61)	n/a	n/a
	HEFI-2019^a^	0.82 (0.80, 0.84)	0.36 (0.30, 0.42)	0.79 (0.76, 0.81)	0.25 (0.18, 0.32)
2020/2021	DQI-I	0.87 (0.86, 0.89)	0.55 (0.50, 0.60)	n/a	n/a
	HEFI-2019^a^	0.83 (0.82, 0.85)	0.41 (0.36, 0.45)	0.80 (0.78, 0.82)	0.29 (0.24, 0.34)
Combined	DQI-I	0.88 (0.87, 0.89)	0.55 (0.52, 0.59)	n/a	n/a
	HEFI-2019^a^	0.83 (0.82, 0.84)	0.38 (0.35, 0.42)	0.80 (0.79, 0.81)	0.29 (0.25, 0.33)

CI, confidence interval; HEI-C 2015, Healthy Eating Index-Canada 2015; DQI-I, Diet Quality Index-International; HEFI-2019, Healthy Eating Food Index 2019; n/a, not applicable. *p*-value for all kappa < 0.01.

a HEFI-2019 scores have been recalibrated from a maximum of 80 to a maximum of 100 by multiplying the scores by 1.25.

Relative to girls, boys reported diets of lower quality, with the difference being particularly pronounced for DQI-I in both univariate (*β* = −1.37, 95% CI: −2.31, −0.43) and multivariable models (β = −1.44, 95% CI: −2.38, −0.50) (Table 3). There were no statistically significant differences in diet quality scores across grade levels regardless of the index used. Students attending schools in more vs. less materially deprived areas appeared to have worse diet quality irrespective of the index used, and these differences remained robust after adjusting for covariates (sex, grade level, energy intake, social deprivation, and geographic region). However, diet quality was higher in more vs. less socially deprived areas when using HEI-C 2015 (*β* = 1.24, 95% CI -0.09, 2.57) and DQI-I (*β* = 1.03, 95% CI: 0.05, 2.02) and after adjusting for covariates (sex, grade level, energy intake, material deprivation, and geographic region). Differences in diet quality according to geographic region were found for HEI-C 2015 but not for DQI-I and HEFI-2019. Finally, energy intake was positively associated with DQI-I (*β* = 0.09, 95% CI: 0.04, 0.14) and negatively associated with HEFI-2019 (β = −0.19, 95% CI: −0.25, −0.12).

**Table 3:** Coefficients (95% CI)^a^ of HEI-C 2015, DQI-I, and HEFI-2019^b^ total scores for participant and school characteristics.

	HEI-C 2015		DQI-I		HEFI-2019^b^	
	Univariate (95% CI)	Multivariable (95% CI)	Univariate (95% CI)	Multivariable (95% CI)	Univariate (95% CI)	Multivariable (95% CI)
Sex (ref: girls)						
Boys	−0.81 (−2.09, 0.47)	−0.69 (−1.96, 0.58)	−1.37 (−2.31, −0.43)^c^	−1.44 (−2.38, −0.50)^c^	−0.84 (−2.14, 0.46)	−0.47 (−1.76, 0.82)
Grade (Ref: 4)						
5	−0.49 (−2.04, 1.07)	−0.22 (−1.78, 1.34)	0.50 (−0.65, 1.65)	0.52 (−0.63, 1.67)	−0.22 (−1.81, 1.36)	0.20 (−1.38, 1.79)
6	1.42 (−0.18, 3.03)	1.44 (−0.18, 3.05)	0.77 (−0.41, 1.96)	0.64 (−0.55, 1.83)	1.22 (−0.41, 2.86)	1.61 (−0.03, 3.25)
Energy intake (per 100 kcal)	0.07 (−0.006, 0.13)	0.04 (−0.03, 0.1)	0.10 (0.06, 0.15)^c^	0.09 (0.04, 0.14)^c^	−0.16 (−0.23, −0.10)^c^	−0.19 (−0.25, −0.12)^c^
Material deprivation (ref: lower)						
Higher	−3.22 (−4.65, −1.78)^c^	−3.05 (−4.52, −1.58)^c^	−1.18 (−2.25, −0.11)^c^	−0.87 (−1.96, 0.22)	−2.03 (−3.50, −0.56)	−2.54 (−4.03, −1.04)^c^
Social deprivation (ref: lower)						
Higher	1.41 (0.12, 2.70)^c^	1.24 (−0.09, 2.57)	1.20 (0.25, 2.15)^c^	1.03 (0.05, 2.02)^c^	−0.24 (−1.55, 1.08)	0.27 (−1.08, 1.62)
Geographic region (ref: urban)						
Rural	1.58 (0.30, 2.86)^c^	1.84 (0.54, 3.14)^c^	0.95 (0.007, 1.89)^c^	0.90 (−0.06, 1.86)	0.79 (−0.51, 2.09)	1.25 (−0.06, 2.57)
Goodness of fit						
F-statistic (*p*-value)		4.24 (<0.01)		6.11 (<0.01)		7.81 (<0.01)
R-squared		0.0172		0.0247		0.0313
Adjusted R-squared		0.0132		0.0207		0.0273
Root MSE		13.159		9.6819		13.331

CI, confidence interval; HEI-C 2015, Healthy Eating Index-Canada 2015; DQI-I, Diet Quality Index-International; HEFI-2019; Healthy Eating Food Index 2019; Root MSE, root mean square deviation.

a From univariate and multivariable linear regression models with variables (sex, grade level, energy intake, material and social deprivation quintiles, and geographic region) added singularly and simultaneously to the models, respectively.

b HEFI-2019 scores have been recalibrated from a maximum of 80 to a maximum of 100 by multiplying the scores by 1.25.

c *p-value < 0.05.*

## 4 Discussion

This study compared diet quality derived using three commonly used summary measures (HEI-C 2015, DQI-I, HEFI-2019) among grade 4–6 students from 25 elementary schools in western Canada. The three indices have different properties (e.g., dietary components assessed, range of values, coefficient of variation), with HEFI-2019 demonstrating the widest range of scores and DQI-I the narrowest variation in the scores. The three indices demonstrated fair to moderate agreement (41). The ability of the indices to discriminate the quality of diets between different population subgroups of Canadian children varied, yielding different conclusions with respect to the determinants of children’s diet quality. Also, higher energy consumption was associated with higher DQI-I and lower HEFI-2019 scores, with the strongest association for HEFI-2019.

This study revealed that, compared with the international index (DQI-I), the two Canadian indices (HEI-C 2015 and HEFI-2019) appear to have more variation in scores. In particular, the index that was developed specifically for Canadian diets (HEFI-2019) showed the widest variation in scores, suggesting it may better capture diets of lower and higher quality. No HEFI-19 scores have been previously reported specifically for children (16). It is therefore not possible to assess whether our scores align with the literature, albeit it is feasible the scores for all three indices may be lower in our sample derived from socioeconomically disadvantaged communities in western Canada. Fair agreement was found between HEI-C 2015 and HEFI-2019 and between DQI-I and HEFI-2019, while a moderate agreement was found between HEI-C 2015 and DQI-I. The latter finding is not surprising as both HEI-C 2015 and DQI-I use similar dietary components (adequacy, moderation) as opposed to HEFI-2019. The correlation between HEI-C 2015 and HEFI-2019 has been previously reported to be 0.79 (16), while in our sample it was as low as 0.6 (data not shown), which may be attributed to our use of a HEI-2015 version that was adapted for the Canadian population (28–30, 34). Finally, higher calorie intake was found to be associated with higher DQI-I but lower HEFI-2019 scores, with HEFI-2019 having the strongest association with energy intake. Brassard et al. also reported the inverse relationship between energy intake and HEFI-2019 scores, proposing that it may be driven by two components which had the highest inverse correlation with energy intake: beverages and vegetables and fruit (16). HEI-C 2015 had no statistically significant association with energy intake in our study. Brassard et al. used the US HEI-2015 and also found no relationship with energy intake since each of its components is divided by total energy intake.

Consistent with government reports (2, 3), our results revealed that boys had worse diet quality than girls regardless of the index used, with DQI-I being the most robust at differentiating diet quality between girls vs. boys. The fact that, unlike HEI-C 2015 and HEFI-2019, DQI-I includes certain nutrients and dietary components (cholesterol, vitamin C, and macronutrient ratio) may have contributed to this finding. Our comparisons across grade levels revealed no statistically significant differences in diet quality regardless of the index used. This could be due to the narrow age range of children in our sample (9–12 years old), whereas previous studies in samples with a wider age range of children demonstrated statistically significant differences (1, 15, 19).

It has been previously demonstrated that children from lower SES families consume less fruit, vegetables and fibre, and more added sugar and energy drinks (42, 43). Our findings corroborate this evidence and show that regardless of the diet quality index used, students from more materially deprived neighbourhoods report worse diet quality, with HEI-C 2015 and HEFI-2019 better capturing these differences. Although diet quality appeared to be higher in more vs. less socially deprived areas, previous studies that reported on the association between social deprivation and diet yielded inconsistent findings (15, 44), possibly due to differences in diet quality indices used, covariates adjusted for, and characteristics of the study sample.

Except when using HEI-C 2015, no difference in diet quality was found between students residing in rural vs. urban areas. Similarly, Tugault et al. reported no significant differences in diet quality between rural vs. urban areas in a sample of 4,728 students 6–17 years old (15). While HEI-C 2015 was able to detect differences in diets between geographic regions, this could be due to smaller sample sizes and surveying participants from smaller geographic areas and no participants from metropolitan areas in our study.

To the best of our knowledge, this is the first study to compare established diet quality indices in a population-based sample of Canadian school-aged children. Data were collected through 24-h dietary recall with a sample size large enough to capture sufficient variation in diet quality across population subgroups. However, there are few limitations to consider. Collecting a single 24-h dietary recall on weekdays does not capture participants’ usual dietary intake; yet collecting data from multiple 24-h dietary recalls and including weekend days is not feasible in school-based studies. The findings of the present study should therefore be interpreted as being based on a single 24-h recall rather than being based on a comparison of usual intake. Since participating schools are located in socioeconomically disadvantaged communities and have an active health promotion intervention in place, children’s diets may differ from those of the general population. However, this does not affect the comparison of the three indices, the indices were able to capture variability in children’s diets as well as variation across population subgroups. All data were self-reported which may be subject to social desirability and measurement bias.

In sum, this study shows that the choice of a diet quality index affects the interpretation of the results and practical considerations. Therefore, researchers, practitioners, and policymakers must seek consensus on which index to use and under which circumstances. Of the three indices examined, HEFI-2019 has been developed most recently specifically for Canadian diets. It reflects adherence to the dietary recommendation outlined in the latest Canada Food Guide and our current understanding of diet quality and how it should be measured. However, adjustments to HEFI-2019 may be needed to circumvent its dependency on diet quantity.

## Data Availability

The data analyzed in this study is subject to the following licenses/restrictions: the raw data supporting the conclusions of this article will be made available by the authors, without undue reservation. Requests to access these datasets should be directed to katerina.maximova@utoronto.ca.

## References

[1] Hack S Jessri M L'Abbe MR. Nutritional quality of the food choices of Canadian children. BMC Nutr. (2021) 7:16. doi: 10.1186/s40795-021-00422-6, PMID: 34049592 PMC8164219

[2] Health Canada. Sodium intake of Canadians in 2017. Ottawa: Health Canada; (2018). Available at: https://www.canada.ca/en/health-canada/services/publications/food-nutrition/sodium-intake-canadians-2017.html

[3] Statistics Canada. Fruit and vegetable consumption, 2017. Ottawa: Statistics Canada (2019) Available at: https://www150.statcan.gc.ca/n1/pub/82-625-x/2019001/article/00004-eng.htm.

[4] Dabravolskaj J Marozoff S Maximova K Campbell S Veugelers PJ. Relationship between fruit and vegetables intake and common mental disorders in youth: a systematic review. Public Health Rev. (2022) 43:1604686. doi: 10.3389/phrs.2022.1604686, PMID: 36204513 PMC9530034

[5] Dalwood P Marshall S Burrows TL McIntosh A Collins CE. Diet quality indices and their associations with health-related outcomes in children and adolescents: an updated systematic review. Nutr J. (2020) 19:118. doi: 10.1186/s12937-020-00632-x, PMID: 33099309 PMC7585689

[6] Peng S Yu Y Yu X Guo D Su L Li H. Adherence to the Chinese dietary guidelines and metabolic syndrome among children aged 6-14 years. Food Funct. (2022) 13:9772–81. doi: 10.1039/d2fo00637e, PMID: 36073110

[7] Florence MD Asbridge M Veugelers PJ. Diet quality and academic performance. J Sch Health. (2008) 78:209–15. doi: 10.1111/j.1746-1561.2008.00288.x 18336680

[8] Sim S Veugelers PJ Prowse R Nykiforuk CI Maximova K. Unhealthy food options in the school environment are associated with diet quality and body weights of elementary school children in Canada. Public Health Nutr. (2021) 24:4572–81. doi: 10.1017/S1368980020004437, PMID: 33143804 PMC10195376

[9] Wu XY Han LH Zhang JH Luo S Hu JW Sun K. The influence of physical activity, sedentary behavior on health-related quality of life among the general population of children and adolescents: a systematic review. PLoS One. (2017) 12:e0187668. doi: 10.1371/journal.pone.0187668, PMID: 29121640 PMC5679623

[10] Setayeshgar S Maximova K Ekwaru JP Gray-Donald K Henderson M Paradis G. Diet quality as measured by the diet quality index-international is associated with prospective changes in body fat among Canadian children. Public Health Nutr. (2017) 20:456–63. doi: 10.1017/S1368980016002500.;, PMID: 27660199 PMC10261485

[11] Hutchinson J Tarasuk V. The relationship between diet quality and the severity of household food insecurity in Canada. Public Health Nutr. (2022) 25:1013–26. doi: 10.1017/S1368980021004031, PMID: 34551845 PMC9991759

[12] Bukambu E Lieffers JRL Ekwaru JP Veugelers PJ Ohinmaa A. The association between the cost and quality of diets of children in Canada. Can J Public Health. (2020) 111:269–77. doi: 10.17269/s41997-019-00264-7, PMID: 31834615 PMC7109244

[13] Gaudin V Stranges S Wilk P Sarma S. School nutrition policy and diet quality of children and youth: a quasi-experimental study from Canada. Can J Public Health. (2023) 114:613–28. doi: 10.17269/s41997-023-00743-y.;, PMID: 36976487 PMC10351299

[14] Lane G Nisbet C Vatanparast H. Dietary habits of newcomer children in Canada. Public Health Nutr. (2019) 22:3151–62. doi: 10.1017/S1368980019001964, PMID: 31387663 PMC10260429

[15] Tugault-Lafleur CN Black JL Barr SI. Examining school-day dietary intakes among Canadian children. Appl Physiol Nutr Metab. (2017) 42:1064–72. doi: 10.1139/apnm-2017-0125, PMID: 28831845

[16] Brassard D Elvidge Munene LA St-Pierre S Guenther PM Kirkpatrick SI Slater J. Development of the healthy eating food index (HEFI)-2019 measuring adherence to Canada's food guide 2019 recommendations on healthy food choices. Appl Physiol Nutr Metab. (2022) 47:595–610. doi: 10.1139/apnm-2021-0415, PMID: 35030038

[17] Gil A Martinez de Victoria E Olza J. Indicators for the evaluation of diet quality. Nutr Hosp. (2015) 31:128–44. doi: 10.3305/nh.2015.31.sup3.8761 25719781

[18] Reedy J Lerman JL Krebs-Smith SM Kirkpatrick SI Pannucci TE Wilson MM. Evaluation of the healthy eating Index-2015. J Acad Nutr Diet. (2018) 118:1622–33. doi: 10.1016/j.jand.2018.05.019.;, PMID: 30146073 PMC6718954

[19] Thomson JL Tussing-Humphreys LM Goodman MH Landry AS. Diet quality in a nationally representative sample of American children by sociodemographic characteristics. Am J Clin Nutr. (2019) 109:127–38. doi: 10.1093/ajcn/nqy284, PMID: 30596813

[20] van der Velde LA Nguyen AN Schoufour JD Geelen A Jaddoe VWV Franco OH. Diet quality in childhood: the generation R study. Eur J Nutr. (2019) 58:1259–69. doi: 10.1007/s00394-018-1651-z.;, PMID: 29516225 PMC6499873

[21] Greene E Murrin C. Parental influences on children's dairy products consumption: a narrative review. Public Health Nutr. (2023) 26:976–93. doi: 10.1017/S1368980022002555, PMID: 36459073 PMC10346067

[22] Manyanga T Tremblay MS Chaput JP Katzmarzyk PT Fogelholm M Hu G. Socioeconomic status and dietary patterns in children from around the world: different associations by levels of country human development? BMC Public Health. (2017) 17:457. doi: 10.1186/s12889-017-4383-8, PMID: 28511721 PMC5434585

[23] Finger JD Varnaccia G Tylleskar T Lampert T Mensink GB. Dietary behaviour and parental socioeconomic position among adolescents: the German health interview and examination survey for children and adolescents 2003-2006 (KiGGS). BMC Public Health. (2015) 15:498. doi: 10.1186/s12889-015-1830-2, PMID: 25985772 PMC4492169

[24] Fung C Kuhle S Lu C Purcell M Schwartz M Storey K. From "best practice" to "next practice": the effectiveness of school-based health promotion in improving healthy eating and physical activity and preventing childhood obesity. Int J Behav Nutr Phys Act. (2012) 9:27. doi: 10.1186/1479-5868-9-27, PMID: 22413778 PMC3414762

[25] Veugelers PJ Dabravolskaj J Khan MKA Tran TT Flynn J Maximova K. From best practice to next practice: implementing comprehensive school health in rural and remote northern communities. Health Promot Chronic Dis Prev Can. (2022) 42:344–52. doi: 10.24095/hpcdp.42.8.04, PMID: 35993604 PMC9514208

[26] Hanning RM Royall D Toews JE Blashill L Wegener J Driezen P. Web-based food behaviour questionnaire: validation with grades six to eight students. Can J Diet Pract Res. (2009) 70:172–8. doi: 10.3148/70.4.2009.172, PMID: 19958572

[27] Willett W. Nutritional epidemiology. 3rd ed. *Monographs in Epidemiology and Biostatistics*. Oxford Academic (2013).

[28] Kennedy ET Ohls J Carlson S Fleming K. The healthy eating index: design and applications. J Am Diet Assoc. (1995) 95:1103–8. doi: 10.1016/S0002-8223(95)00300-2, PMID: 7560680

[29] Anic GM Park Y Subar AF Schap TE Reedy J. Index-based dietary patterns and risk of lung cancer in the NIH-AARP diet and health study. Eur J Clin Nutr. (2016) 70:123–9. doi: 10.1038/ejcn.2015.122, PMID: 26264348

[30] Zhang Y Lu C Li X Fan Y Li J Liu Y. Healthy eating Index-2015 and predicted 10-year cardiovascular disease risk, as well as heart age. Front Nutr. (2022) 9:888966. doi: 10.3389/fnut.2022.888966, PMID: 35903444 PMC9315384

[31] Glanville NT McIntyre L. Diet quality of Atlantic families headed by single mothers. Can J Diet Pract Res. (2006) 67:28–35. doi: 10.3148/67.1.2006.28, PMID: 16515745

[32] Woodruff SJ Hanning RM. Development and implications of a revised Canadian healthy eating index (HEIC-2009). Public Health Nutr. (2010) 13:820–5. doi: 10.1017/S1368980009993120, PMID: 20074391

[33] Garriguet D. Diet quality in Canada. Health Rep. (2009) 20:41–52. PMID: 19813438

[34] Jessri M Ng AP L'Abbe MR. Adapting the healthy eating index 2010 for the Canadian population: evidence from the Canadian National Nutrition Survey. Nutrients. (2017) 9:910. doi: 10.3390/nu9080910, PMID: 28825674 PMC5579703

[35] Marozoff S Veugelers PJ Dabravolskaj J Eurich DT Ye M Maximova K. Diet quality and health service utilization for depression: a prospective investigation of adults in Alberta's tomorrow project. Nutrients. (2020) 12:2437. doi: 10.3390/nu12082437, PMID: 32823652 PMC7468802

[36] Health Canada. Eating well with Canada's food guide. Ottawa: Health Canada (2007).

[37] Kim S Haines PS Siega-Riz AM Popkin BM. The diet quality index-international (DQI-I) provides an effective tool for cross-national comparison of diet quality as illustrated by China and the United States. J Nutr. (2003) 133:3476–84. doi: 10.1093/jn/133.11.3476, PMID: 14608061

[38] Pampalon R Raymond G. A deprivation index for health and welfare planning in Quebec. Chronic Dis Can. (2000) 21:104–13. PMID: 11082346

[39] Statistics Canada. Population Centre and rural area classification 2016. Ottawa: Ontario (2017) Available at: https://www.statcan.gc.ca/eng/subjects/standard/pcrac/2016/introduction.

[40] Sim J Wright CC. The kappa statistic in reliability studies: use, interpretation, and sample size requirements. Phys Ther. (2005) 85:257–68. doi: 10.1093/ptj/85.3.257, PMID: 15733050

[41] Dettori JR Norvell DC. Kappa and beyond: is there agreement? Global Spine J. (2020) 10:499–501. doi: 10.1177/2192568220911648, PMID: 32435572 PMC7222679

[42] Minaker LM McCargar L Lambraki I Jessup L Driezen P Calengor K. School region socio-economic status and geographic locale is associated with food behaviour of Ontario and Alberta adolescents. Can J Public Health. (2006) 97:357–61. doi: 10.1007/BF03405342, PMID: 17120872 PMC6975837

[43] Thomas F Thomas C Hooper L Rosenberg G Vohra J Bauld L. Area deprivation, screen time and consumption of food and drink high in fat salt and sugar (HFSS) in young people: results from a cross-sectional study in the UK. BMJ Open. (2019) 9:e027333. doi: 10.1136/bmjopen-2018-027333, PMID: 31256025 PMC6609085

[44] Fismen AS Buoncristiano M Williams J Helleve A Abdrakhmanova S Bakacs M. Socioeconomic differences in food habits among 6- to 9-year-old children from 23 countries-WHO European childhood obesity surveillance initiative (COSI 2015/2017). Obes Rev. (2021) 22:e13211. doi: 10.1111/obr.13211, PMID: 34235830

