# Supplementary material for: Comparing three summary indices to assess diet quality of Canadian children: a call for consensus
Source: Front Nutr. 2025 Jan 7;11:1519829. doi: 10.3389/fnut.2024.1519829 (PMC11746050; doi:10.3389/fnut.2024.1519829)
Supplement: Supplementary file 1 [file Data_Sheet_1.PDF]

Supplementary Material

Comparing three summary indices to assess diet quality of Canadian children: a call for consensus

Seyedehatefeh Panahimoghadam, Paul J. Veugelers, Julia Dabravolskaj, Trudy Tran, Katerina Maximova*

*** Correspondence:** Katerina Maximova: katerina.maximova@utoronto.ca

1 Supplementary Table

Components and scoring standards for HEI-C 2015, DQI-I, HEFI-2019

Category (points)	Component (points)	Measurement Unit	Maximum score	Minimum score
		**HEI-C 2015**		
Adequacy (60)	Total Fruits and Vegetables (10)	Servings/d	6 servings	No Fruits and Vegetables
	Whole Fruits (5)	Servings/d	1.26 servings	No Whole Fruits
	Greens & Beans (5)	Servings/d	0.63 servings	No Dark Green Vegetables or Legumes
	Whole Grains (10)	Servings/d	3 servings	No Whole Grains
	Total Protein Foods (5)	Servings/d	1-2 servings	No Protein Foods
	Seafood & Plant Proteins (5)	Servings/d	0.32-0.64 servings	No Seafood or Plant Proteins
	Dairy (10)	Servings/d	3-4 servings	No Dairy
	Fatty Acids (10)	(PUFA + MUFA)/SFA	≥2.5	≤1.2
Moderation (40)	Refined Grains (10)	Total refined grain/ Total grain foods	<50%	100%
	Added Sugars (10)	Of total energy/d	≤6.5%	≥26%
	Saturated Fats (10)	Of total energy/d	≤8%	≥16%
	Sodium (10)	UL (mg/d)	≤UL (≤2200 mg/d)	≥2xUL (≥4400)
		**DQI-I**		
Adequacy (40)	Vegetable group (5)	Servings/d	≥3-5	0
	Fruit group (5)	Servings/d	≥ 2–4	0
	Fiber (5)	DRI (g/d)	≥20-30	0
	Grain group (5)	Servings/d	≥6-11	0
	Protein (5)	of total energy/d	≥10%	0%
	Iron (5)	RDA (AI)/d	≥100%	0%
	Calcium (5)	AI/d	≥100%	0%

	Vitamin C (5)	RDA (RNI)/d	≥100%	0%
Moderation (30)	Total fat (6)	Of total energy/d	≤20%	>30%
	Cholesterol (6)	mg/d	≤300	>400
	Saturated fats (6)	Of total energy/d	≤7%	>10%
	Empty calorie foods (6)	Of total energy/d	≤3%	>10%
	Sodium (6)	mg/d	≤2400	>3400
Variety (20)	Overall food group variety (meat, poultry, fish, egg; dairy, beans; grain; fruit; vegetable) (15)	Servings from food groups/d	≥1 serving from each food group/d	None from any food groups
	Within-group variety for protein source (meat, poultry, fish, dairy, beans, eggs) (5)	Number of different sources/d	≥3 different sources/d	None
Overall balance (10)	Fatty acid ratio (PUFA:MUFA:SFA) (4)	P/S and M/S	P/S = 1-1.5 and M/S = 1-1.5	Otherwise
	Macronutrient ratio (carbohydrate:protein:fat) (6)	Carbohydrate:protein:fat	55-65:10-15:15-25	
		**HEFI-2019**		
Foods (40)	Vegetables and fruits (20)	Total vegetables and fruits/Total foods (RA/RA)	≥0.50	No fruits and vegetables
	Whole grain foods (5)	Total whole grain foods/Total foods (RA/RA)	≥0.25	No whole-grain foods
	Grain foods ratio (5)	Total whole-grain foods/Total grain foods (RA/RA)	1	No whole-grain foods
	Protein foods (5)	Total protein foods/Total foods (RA/RA)	≥0.25	No Protein foods
	Plant-based protein foods (5)	Plant-based protein foods/Total protein foods (RA/RA)	≥0.5	No plant-based protein foods
Nutrients (30)	Saturated fats (5)	Total saturated fat/energy (kcal/kcal)	<10%	≥15%
	Free sugars (10)	Total free sugars / energy (kcal/kcal)	<10%	≥20%
	Sodium (10)	Total sodium / energy (mg/kcal)	<0.9	≥2.0
	Fatty acids ratio (5)	(Mono + polyunsaturated fat)/Total saturated fat (g/g)	≥2.6	≤1.1
Beverages (10)	Beverages (10)	(Plain water including carbonated + unsweetened beverages)/Total beverages (g/g)	1	No water and no unsweetened beverages

Abbreviations: HEI-C 2015, Healthy Eating Index-Canada 2015; DQI-I, Diet Quality Index-International; HEFI-2019, Healthy Eating Food Index 2019; RA, Reference Amounts; DRI, Dietary Reference Intakes; RDA, Recommended Dietary Allowance; AI, Adequate Intakes; RNI, Recommended Nutrient Intake; PUFA, polyunsaturated fatty acids; MUFA, monounsaturated fatty acids; SFA, saturated fatty acids; UL, Tolerable Upper Intake Level.
